# Mitofusin-2 is required for mouse oocyte meiotic maturation

**DOI:** 10.1038/srep30970

**Published:** 2016-08-03

**Authors:** Jing-Hua Zhang, Teng Zhang, Si-Hua Gao, Ke Wang, Xiu-Yan Yang, Fang-Fang Mo, Na Yu, Tian An, Yu-Feng Li, Ji-Wei Hu, Guang-Jian Jiang

**Affiliations:** 1 Cancer Institute, Tangshan People’s Hospital, Tang Shan, China; 2 State Key Laboratory of Stem Cell and Reproductive Biology, Institute of Zoology, Chinese Academy of Sciences, Beijing, China; 3 Diabetes research Center of Beijing University of Chinese Medicine, Beijing, China; 4 Central Laboratory, Cancer Institute, Tangshan People’s Hospital, Tang Shan, China

## Abstract

Mitofusin-2 (*Mfn2*) is essential for embryonic development, anti-apoptotic events, protection against free radical-induced lesions, and mitochondrial fusion in many cells. However, little is known about its mechanism and function during oocyte maturation. In this study, we found that *Mfn2* was expressed in the cytoplasm during different stages of mouse oocyte maturation. *Mfn2* was mainly associated with α-tubulin during oocyte maturation. Knockdown of *Mfn2* by specific siRNA injection into oocytes caused the mitochondrial morphology and quantity to change, resulting in severely defective spindles and misaligned chromosomes. This led to metaphase I arrest and the failure of first polar body extrusion. Furthermore, *Mfn2* depletion from GV stage oocytes caused the redistribution of p38 MAPK in oocyte cytoplasm. These findings provide insights into potential mechanisms of *Mfn2*-mediated cellular alterations, which may have significant implications for oocyte maturation.

The mitofusin-2 (*Mfn2*) gene, also called the hyperplasia suppressor gene, was identified by screening vascular smooth muscle cell cDNA libraries of Wistar–Kyoto and spontaneously hypertensive rats using the differential display technique. *Mfn2* is a powerful suppressor of cell proliferation *in vivo* and *in vitro*. The anti-proliferative effect of *Mfn2* is mediated by inhibition of ERK/mitogen-activated protein kinase (MAPK) signalling and subsequent cell-cycle arrest^1^.

Mitochondria are the most abundant organelles in mammalian oocytes and early embryos^2^. Because mitochondria produce ATP through aerobic respiration, they became a driving force in evolution^3^. Optimal mitochondrial function is ensured by a quality-control system tightly coupled to fusion and fission. The fusion of mitochondria plays a crucial role in embryonic development^4^. *Mfn2* participates in mitochondrial fusion and plays an essential role in metabolic homeostasis^5^,^6^. Increasing evidence indicates that *Mfn2* not only plays critical roles in energy metabolism, endoplasmic reticulum stress, and signal transduction, but also has a close relationship with blastocyst formation and early embryonic development^7^,^8^,^9^,^10^,^11^. However, the role and mechanism of *Mfn2* in regulating oocyte maturation is still unknown.

Oocyte maturation is a complex and precisely synchronized process affected by many factors. Oocyte maturation refers to the meiotic process that takes place from the germinal vesicle (GV) stage to the metaphase II stage. The first indication for this process is the disappearance of the GV as observed under the light microscope. This change is called germinal vesicle breakdown (GVBD). After GVBD, oocytes pass through metaphase of the first meiotic division and entry into the second meiotic division. Thereafter, oocytes are arrested at metaphase of the second meiotic division until fertilization takes place^12^.

Cell division involves precise spindle organization and chromosome segregation. Functional analysis of p38 MAPK in mouse oocytes suggests that this kinase regulates spindle assembly and accurate chromosome segregation through phosphorylation of MAPK-activated protein kinase^13^,^14^. In porcine oocytes, p38 MAPK contributes to the transition of metaphase I to metaphase II^15^. Previous study showed that Mfn2 could affect p38 mitogen-activated protein kinases (MAPKs) pathway and have relationship with p38 MAPK phosphorylation in somatic cells^16^,^17^. However, the interactions remain unclear between *Mfn2* and p38 MAPK during oocyte maturation.

Here, we demonstrate that *Mfn2* is indispensable for the meiotic progression and mitochondrial morphology as well as the localization of p38 MARK in mouse oocytes.

## Results

### Expression and subcellular localization of *Mfn2* during oocyte meiotic maturation

**Figure 1:**
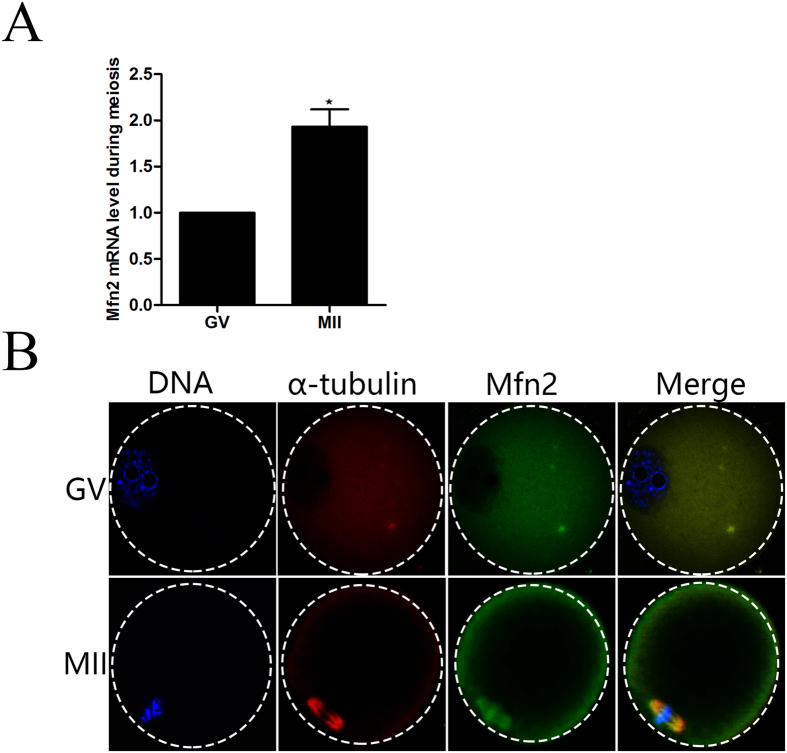
Expression and subcellular localization of *Mfn2* during mouse oocyte meiotic maturation. (**A**) Expression of *Mfn2* mRNA was determined by qT-PCR analysis. (**B**) Confocal microscopy showing the subcellular localization of *Mfn2* (green), α-tubulin (red), and DNA (blue) in mouse oocytes at the GV and MII stages.

To investigate the role of *Mfn2* during meiosis, the expression and subcellular localization of this protein was examined. Expression of *Mfn2* in GV and ovulated MII oocytes was detected by the quantitative real-time polymerase chain reaction (qRT-PCR). The mRNA level of *Mfn2* was moderate at GV stages, and reached the highest level at MII stages (Fig. 1A) (P < 0.05). To assess the subcellular location of *Mfn2* protein during meiotic maturation, GV and ovulated MII mouse oocytes were immunolabelled with anti-α-tubulin and anti-*Mfn2* antibodies to visualize the spindle and *Mfn2*, respectively. Oocytes were co-stained with Hoechst 33342 to visualize the chromosomes. In GV oocytes, *Mfn2* was distributed mainly in the cytoplasm. Interestingly, *Mfn2* accumulated in the sub-membrane region and was concentrated in the region of the spindle and chromosomes in MII oocytes (Fig. 1B).

### Knockdown of *Mfn2* causes MI arrest and reduces PB1 extrusion

**Figure 2:**
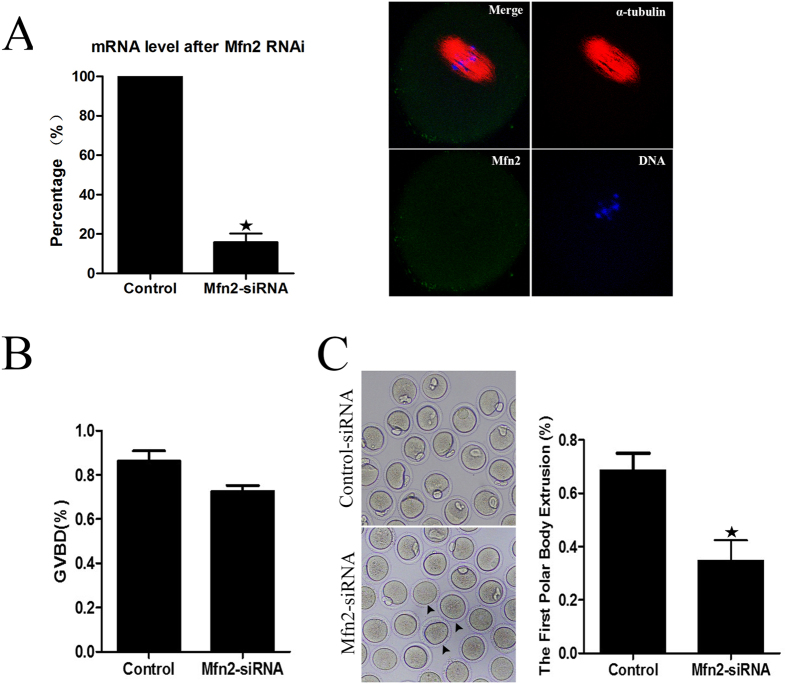
*Mfn2* siRNA inhibits the GVBD and first polar body extrusion. (**A**) Levels of *Mfn2* mRNA and protein in oocytes injected with siRNA. (**B**) Percentages of GVBD in siRNA-injected and control oocytes. (**C**) Percentages of first polar body extrusion in siRNA-injected and control oocytes. Data are expressed as means ± SEM of at least three independent experiments. *Significantly different from control (p < 0.05).

To determine whether *Mfn2* functions in regulating mouse oocyte maturation, *Mfn2*-siRNA was microinjected into the cytoplasm of GV oocytes. The efficiency of siRNA was determined by measuring *Mfn2* mRNA levels by qRT-PCR. Compared with the control, the mRNA level of *Mfn2* in siRNA- injected oocytes was significantly decreased (15.8 ± 7.7 vs. 100%, n = 150, p < 0.05). Immunofluorescent staining results showed that, after RNAi, there was minimal specific localization of *Mfn2* around spindles, implying successful *Mfn2* downregulation by siRNA (Fig. 2A). Compared with the control, the extent of GVBD in siRNA- injected oocytes was significantly decreased (86.3 ± 8.03 vs. 72.7 ± 4.35%, n = 186, p < 0.05, Fig. 2B). After culturing for 14 h, the extrusion of PB1 in the siRNA group (34.9 ± 13.1%, n = 152) was considerably lower than that in the control group (68.7 ± 10.9%, n = 158, p < 0.05, Fig. 2C).

### Depletion of *Mfn2* induces spindle defects and chromosome misalignment during oocyte maturation

**Figure 3:**
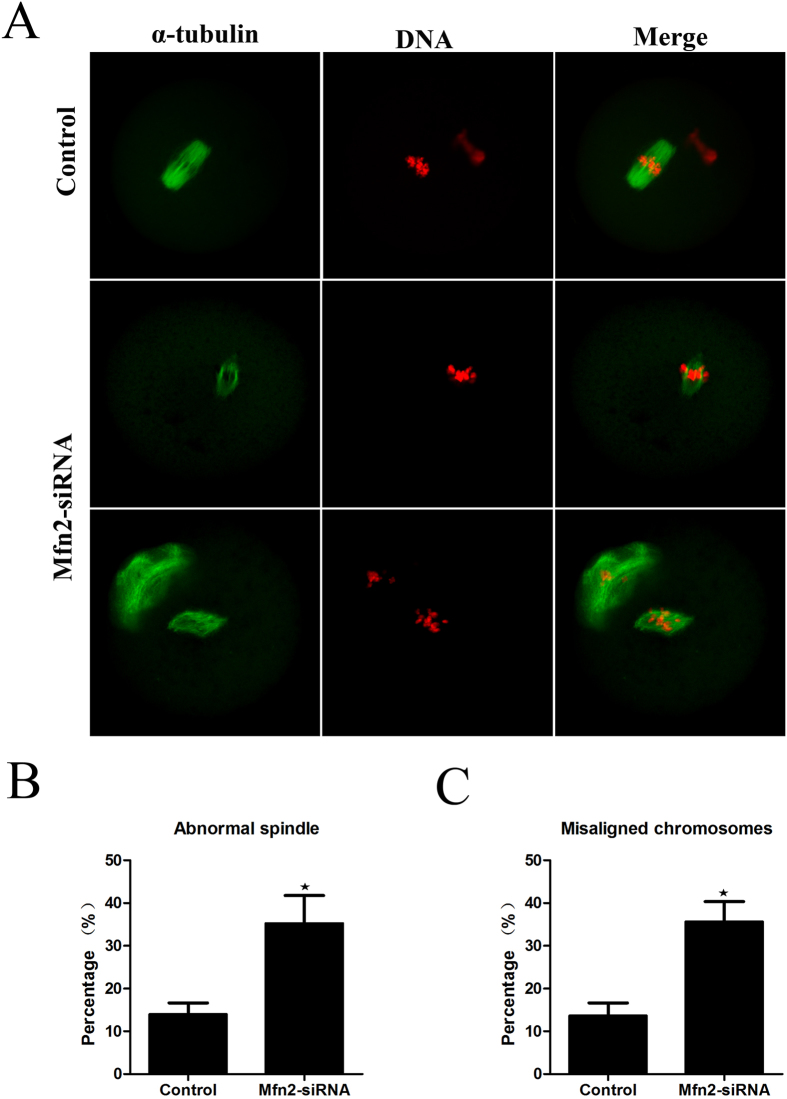
*Mfn2* siRNA causes defects of the meiotic spindle and chromosome alignment. (**A**) Oocytes microinjected with *Mfn2* or control siRNA were incubated in M2 medium containing 2.5 μM milrinone for 24 h, and then transferred to milrinone-free M2 for 12 h. This was followed by immunostaining with an α-tubulin antibody (green) and Hoechst 33342 (red). (**B**,**C**) The percentages of oocytes with abnormal spindles or misaligned chromosomes in siRNA-injected and control oocytes. Data are expressed as means ± SEM of at least three independent experiments. *Significantly different from control (p < 0.05).

To explore the possible effects of *Mfn2* on the organization of the spindle and chromatin during meiotic maturation, we examined spindle morphology and chromosome distribution by using confocal microscopy. As shown in Fig. 3A, downregulation of *Mfn2* resulted in significant defects in spindle formation and chromosome alignment. The extent of abnormal spindle formation in the experiment group was (35.6 ± 11.4%) (n = 172), which was considerably higher than the control group (14.4 ± 4.86%, p < 0.05) (n = 168) (Fig. 3B). *Mfn2*-depleted oocytes displayed severe defects in chromosome alignment, showing lagging and irregularly scattered chromosomes (Fig. 3A). The incidence of misaligned chromosomes in the experimental group was 35.8 ± 7.99% (n = 172), much higher than the control group (14.1 ± 5.44%, p < 0.05) (n = 168) (Fig. 3C). These data suggest that *Mfn2* is required for regulating spindle organization and chromosome alignment.

### Downregulation of *Mfn2* causes the redistribution of mitochondria

**Figure 4:**
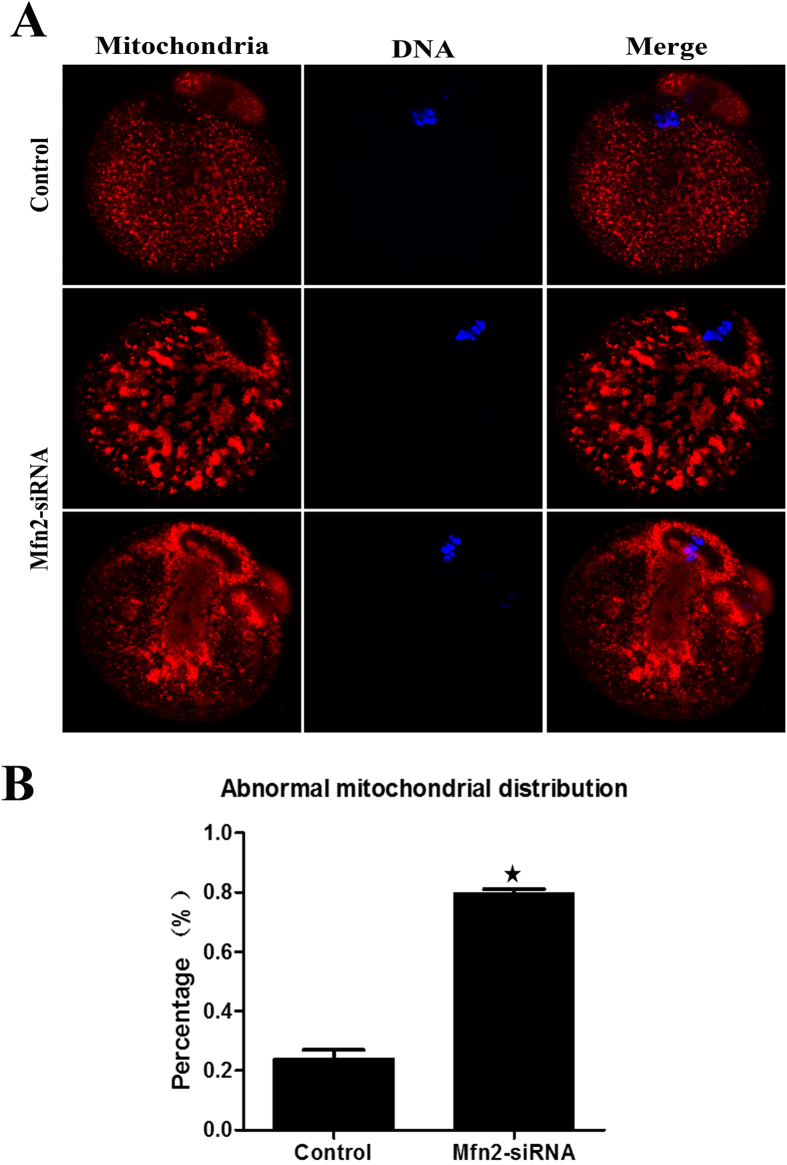
*Mfn2* siRNA causes the redistribution of mitochondria. (**A**) Oocytes microinjected with *Mfn2* or control siRNA were incubated in M2 medium containing 2.5 μM milrinone for 24 h, and then transferred to milrinone-free M2 medium for 12 h. This was followed by immunostaining with MitoTracker (red) and Hoechst 33342 (blue). (**B**) The percentages of oocytes with abnormal distribution of mitochondria in the *Mfn2* siRNA and control groups. Data are expressed as means ± SEM of at least three independent experiments. *Significantly different from control (p < 0.05).

In order to determine whether *Mfn2* influences the spatial remodelling of mitochondria during oocyte maturation, we compared the mitochondrial distribution patterns between oocytes from *Mfn2*-depleted and control oocytes. A homogeneous distribution of mitochondria throughout the entire ooplasm was observed by immunofluorescence microscopy in control oocytes (Fig. 4A). Increased mitochondrial clustering was readily observed in *Mfn2*-depleted oocytes (79.6 ± 2.46%, n = 126) as compared with control oocytes (24.1 ± 5.06%, n = 122, P < 0.05) (Fig. 4B). These results suggest that depletion of *Mfn2* can disrupt mitochondrial distribution during oocyte maturation.

### Depletion of *Mfn2* causes the location of p38 MAPK to become scattered

**Figure 5:**
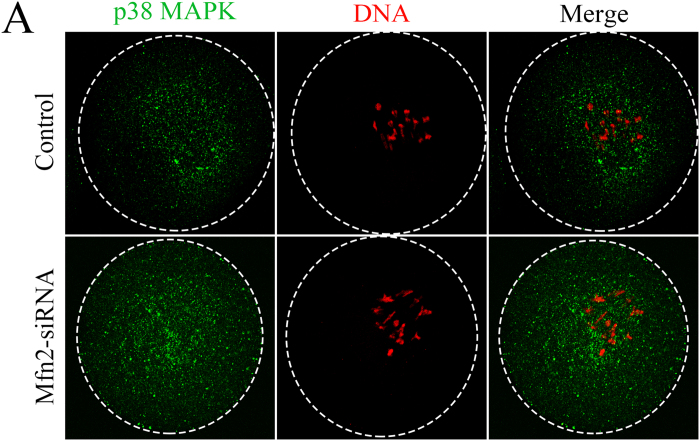
Changes in the distribution of p38 MAPK in oocytes injected with *Mfn2* or control siRNA. Confocal microscopy showing the subcellular localization of p38 MAPK (green) and DNA (red) in oocytes injected with *Mfn2* or control siRNA.

To further investigate the mechanism and signal transduction pathway of *Mfn2* during oocyte meiotic maturation, we used a specific *Mfn2*-siRNA to deplete most of the endogenous *Mfn2* in oocytes. Immunofluorescence microscopy showed that p38 MAPK was scattered in an irregular pattern in the cytoplasm of oocytes after *Mfn2* depletion. In contrast, in control oocytes, p38 MAPK accumulated mainly in the spindle region (Fig. 5). This correlation strongly suggests that a deficiency of *Mfn2* may be linked directly to abnormal p38 MAPK distribution that then retards oocyte maturation.

## Discussion

Increasing data imply that *Mfn2* may play critical roles in female mammalian reproduction^18^,^19^. In the current study, we show that *Mfn2* is expressed in mouse oocytes from the GV and MII stages (Fig. 1A). In mouse oocytes, the subcellular localization of *Mfn2* was obvious at the spindle at the meiotic metaphase. The localization of *Mfn2* completely overlapped with that of spindle at the meiotic metaphase stages (Fig. 1B), suggesting that *Mfn2* may participate in the spindle formation during meiotic maturation. The localization during oocyte meiosis was similar to that in previous study^11^. GVBD is a step in the development of oocytes, marking their maturation. Our data show that depletion of *Mfn2* from the GV oocyte leads to less GVBD, less extrusion of the PB1, and developmental retardation. Our study also shows that, in MII oocytes, *Mfn2* locates to the spindle, binds to microtubules, and functions as a major factor of meiotic spindle assembly and chromosome segregation. We extended our studies to examine spindle and chromosome organization during oocyte meiosis. Knockdown of *Mfn2* in meiotic oocytes led to spindle organization defects and chromosome misalignment, thus leading to cell cycle arrest at the MI phase. These results suggest that the main function of *Mfn2* in meiosis is to organize microtubules to form the spindle and segregate the chromosomes.

Early studies suggested that microtubules were the major component of cytoskeletal systems responsible for regulating the distribution of mitochondria in mammalian cells^20^,^21^. This finding compelled us to consider the possibility of microtubule-mitochondria binding at the level of protein-protein interactions. Several studies in mammalian species have shown that mitochondria undergo stage-specific changes in distribution during oocyte maturation^22^. Such a spatial remodelling of mitochondria may allow maturing oocytes to cater to differing energy requirements and provide a means for environmental sensing. In our study, the normal distribution pattern of mitochondria during meiotic maturation was disrupted in *Mfn2*-depleted oocytes resulting in clustered mitochondria. The spatial remodelling of mitochondria during oocyte maturation may reflect different ATP requirements at different developmental stages^23^.

Mammalian oocytes undergo intracytoplasmic mitochondria translocation during maturation and fertilization. This translocation is a microtubule-mediated cellular event. It is generally thought that mitochondrial spatial remodelling may be indicative of the energy requirement of various key events, such as GVBD and metaphase spindle formation^23^. Therefore, inadequate redistribution of mitochondria may be an important factor contributing to maturation delay and spindle/chromosome disorganization. The redistribution and changeability of mitochondria suggests that, in addition to its function in regulating mitochondrial fusion, *Mfn2* is involved in other roles that control mitochondrial morphology and distribution.

These findings prompted us to investigate the effects of *Mfn2* on the status of mitochondria, and spindle and chromosome organization in mouse oocytes, and then explore the relationship between these effects. Previously, the regulation of meiotic spindle organization and chromosome segregation in mammalian oocytes has not been studied as well as that in mitotic somatic cells. In the current study, we found a higher percentage of chromosome failure, spindle shape changes, and mitochondrial clustering in *Mfn2* knockdown oocytes, suggesting this as a cause of oocyte maturation retardation. This correlation strongly suggests that deficient chromosome alignment may be directly linked to abnormal mitochondrial distribution, and is consistent with previous reports showing the involvement of mitochondrial function in spindle assembly and genomic stability of germ cells^24^,^25^.

*Mfn2* plays an important role in spindle integrity and cell cycle progression in meiotic oocytes. p38 MAPK is one of the MAPKs involved in cell differentiation and apoptosis. p38 MAPK is a microtubule-associated protein and is required for stabilizing spindle assembly in mouse oocytes. p38α is required for the recruitment of γ-tubulin to the microtubule organizing centre and stabilization of spindle bipolarity. Depletion of p38α may compromise meiotic spindle organization and chromosome alignment via the p38α/MAPK-activated protein kinase signalling pathway.

Although MAPKs have been implicated in oocyte maturation in several species^26^,^27^, there is very limited information regarding the relationship between p38 MAPK and *Mfn2* during oocyte meiosis. In the current study, the function of *Mfn2* in oocyte maturation was found to rely on the p38-MAPK signalling pathways as shown by our findings of abnormal localization and expression of p38-MAPK after microinjecting *Mfn2* siRNA into GV oocytes. Collectively, our observations indicated that *Mfn2* was present in different oocyte developmental stages. *Mfn2* knockdown oocytes displayed a higher frequency of spindle defects and chromosome misalignment in meiosis. *Mfn2* affected the maturation of mammalian oocytes, possibly through changes in mitochondrial function and the p38 MAPK signalling pathway.

In general, *Mfn2* dysfunction has emerged as a key factor in a myriad of diseases and metabolic disorders^28^,^29^,^30^,^31^,^32^,^33^. The current study provides new information about how *Mfn2* functions impinge on mouse oocyte maturation. Further studies are needed to elucidate the precise mechanism and biological significance of *Mfn2* in oocyte maturation.

## Materials and Methods

### Ethics statement

This study was approved by the Animal Care and Use Committee of the Institute of Zoology, Chinese Academy of Sciences. All animal manipulations were performed according to the guidelines of the Animal Care and Use Committee.

### Collection and culture of mouse oocytes

Oocytes were collected from 4–6 week-old ICR mice. To obtain GV oocytes, females were primed with 5 IU of pregnant mare serum gonadotropin and sacrificed after 48 h. By puncturing the fully grown follicles, GV oocytes were released from the ovaries into pre-warmed M2 medium. MII oocytes were collected as described previously^34^,^35^. After specific treatments, oocytes were washed thoroughly and cultured in M2 medium, undergoing GV, GVBD, MI, and MII stages.

### Immunofluorescence analysis

Oocytes were washed three times with phosphate-buffered saline (PBS) and then fixed in 4% paraformaldehyde in PBS at 4 °C for 1 h. Fixed cells were washed 3 times with PBST (PBS supplemented with 0.1% Tween 20) and incubated in 0.1% Triton X-100 in PBS at room temperature for 30 min. After washing with PBST, oocytes were blocked with 5% bovine serum albumin in PBS at room temperature for 1 h and transferred into diluted media containing a rabbit anti-*Mfn2* antibody (1:50, a kind gift of Professor Chen Kuang-Hueih), a monoclonal anti-tubulin antibody (1:100), or a rabbit anti-p-p38 antibody (1:100) either at room temperature for 2 h or overnight at 4 °C. Finally, the labelled oocytes were washed with PBST and stained with FITC-labelled goat-anti-rabbit IgG (1:100) and TRITC-labelled goat-anti-mouse IgG (1:100) (Santa Cruz Biotechnology, Santa Cruz, CA, USA) at room temperature for 2 h. For mitochondrial staining, oocytes were incubated for 30 min at 37 °C in M2 medium supplemented with 200 nM MitoTracker Red. The oocytes were finally stained with Hoechst 333342 after three washes in washing buffer and were mounted on glass slides for immunofluorescence microscopy. Photos were captured using a confocal laser-scanning microscope (Zeiss LSM 780, Oberkochen, Germany).

### siRNA microinjection

All injections were carried out according to the procedures described previously^36^. The small interfering RNA (siRNA) of *Mfn2* (sequence: UCCUCAAGGUUUAUAAGAATT) (GenePharma, Shanghai, China), or the siRNA control, was microinjected (25 μM) into the cytoplasm of fully grown GV oocytes with an Eppendorf microinjection instrument (Hamburg, Germany) and completed within 30 min. Oocytes were kept in M2 medium supplemented with 2.5 μM milrinone (Sigma-Aldrich, St. Louis, MO, USA) to prevent GV breakdown and to allow *Mfn2*-siRNA to complete its role during this period. After 24 h, the oocytes were cleaned thoroughly to resume meiosis. Each experiment was repeated three to five times.

### qRT-PCR

Total RNA was extracted from GV and MII mouse oocytes using an RNeasy micro-purification kit (Qiagen, Valencia, CA, USA) and then reverse transcribed to cDNA with an oligo dT primer using a Prime Script 1st Strand cDNA Synthesis Kit (TaKaRa Bio, Shiga, Japan). The full length *Mfn2* coding sequence was amplified by PCR with the following primers: Forward: 5′-CCCCTGGCTCATACCCTAAT-3′, Reverse: 5′-AAGTAGGAGTGGCTGCCTGA-3′. Actin was selected as the reference gene. The SYBR Premix Ex Tag2 kit (TaKaRa Bio) was used in an ABI Prism 7500 sequence detection system (Life Technologies, Carlsbad, CA, USA). Relative gene expression was calculated by the 2^ΔΔCt^ method.

### Statistical analysis

Data (means ± SEM) were from at least three replicates per experiment and analysed by ANOVA using SPSS software (IBM, Chicago, IL, USA) followed by Fisher’s LSD test. Differences at p < 0.05 were considered to be statistically significant

## Additional Information

**How to cite this article**: Zhang, J.-H. *et al*. Mitofusin-2 is required for mouse oocyte meiotic maturation. *Sci. Rep.* **6**, 30970; doi: 10.1038/srep30970 (2016).
